# Microbiological and Geochemical Survey of CO_2_-Dominated Mofette and Mineral Waters of the Cheb Basin, Czech Republic

**DOI:** 10.3389/fmicb.2017.02446

**Published:** 2017-12-11

**Authors:** Patryk Krauze, Horst Kämpf, Fabian Horn, Qi Liu, Andrey Voropaev, Dirk Wagner, Mashal Alawi

**Affiliations:** ^1^GFZ German Research Centre for Geosciences, Section 5.3 Geomicrobiology, Potsdam, Germany; ^2^GFZ German Research Centre for Geosciences, Section 3.2 Organic Geochemistry, Potsdam, Germany; ^3^Hydroisotop GmbH, Schweitenkirchen, Germany; ^4^Institute for Earth and Environmental Sciences, University of Potsdam, Potsdam, Germany

**Keywords:** elevated CO_2_ concentration, microbial ecology, deep biosphere, Eger Rift, paleo-sediment, *Sulfuricurvum*, *Gallionella*, *Sideroxydans*

## Abstract

The Cheb Basin (NW Bohemia, Czech Republic) is a shallow, neogene intracontinental basin. It is a non-volcanic region which features frequent earthquake swarms and large-scale diffuse degassing of mantle-derived CO_2_ at the surface that occurs in the form of CO_2_-rich mineral springs and wet and dry mofettes. So far, the influence of CO_2_ degassing onto the microbial communities has been studied for soil environments, but not for aquatic systems. We hypothesized, that deep-trenching CO_2_ conduits interconnect the subsurface with the surface. This admixture of deep thermal fluids should be reflected in geochemical parameters and in the microbial community compositions. In the present study four mineral water springs and two wet mofettes were investigated through an interdisciplinary survey. The waters were acidic and differed in terms of organic carbon and anion/cation concentrations. Element geochemical and isotope analyses of fluid components were used to verify the origin of the fluids. Prokaryotic communities were characterized through quantitative PCR and Illumina 16S rRNA gene sequencing. Putative chemolithotrophic, anaerobic and microaerophilic organisms connected to sulfur (e.g., *Sulfuricurvum, Sulfurimonas*) and iron (e.g., *Gallionella, Sideroxydans*) cycling shaped the core community. Additionally, CO_2_-influenced waters form an ecosystem containing many taxa that are usually found in marine or terrestrial subsurface ecosystems. Multivariate statistics highlighted the influence of environmental parameters such as pH, Fe^2+^ concentration and conductivity on species distribution. The hydrochemical and microbiological survey introduces a new perspective on mofettes. Our results support that mofettes are either analogs or rather windows into the deep biosphere and furthermore enable access to deeply buried paleo-sediments.

## Introduction

Mofettes are cold (≤30°C) natural exhalations of magmatogene carbon dioxide, which migrates through the lithospheric upper mantle and crust through surface water and soil to the atmosphere (Pfanz, 2008; Kämpf et al., 2013). Such CO_2_ vents are located both in volcanically active and in non-volcanic, but seismically active regions (Evans et al., 2002; Lan et al., 2007). The cold exhalations of mantle-derived carbon dioxide occur as “wet mofettes” (CO_2_ runs/bubbles through surface water) and “dry mofettes” (CO_2_ migrates through subsurface sediments and soil; Kämpf et al., 2013). Both types show diffuse and channelized CO_2_ degassing. Mofettes are local degassing phenomena, but also integral parts of larger Diffuse Degassing Structures (DDS) on the scale of up to a few square kilometers, controlled by the fluid migration inside of fault zones (e.g., Girault and Perrier, 2014; Nickschick et al., 2015) or in volcano-hydrothermal areas (e.g., Chiodini et al., 2008; Girault et al., 2014). Recently, mofettes were used as model ecosystems for studying the response of soil microbiota to potential CO_2_ leakage from underground carbon capture and storage systems (e.g., Krüger et al., 2009, 2011; Frerichs et al., 2013).

At the surface, the dry mofettes show a characteristic vegetation which is at some sites strongly declined or even completely missing. Dry mofettes draw attention as an extreme habitat in ecological research investigating the response of plants (Vodnik et al., 2002), arbuscular mycorrhizal fungi (Maček et al., 2011) or soil archaea and bacteria (Šibanc et al., 2014) to elevated CO_2_ concentrations. Generally, a shift to anaerobic/microaerophilic and acidophilic community compositions has been reported for mofette soils compared to respective control sites. Bacterial community richness, evenness and diversity decreased with increasing CO_2_ flux (Sáenz de Miera et al., 2014). The abundance of methanogenic archaea and sulfate reducing bacteria, which could use geogenic CO_2_ for assimilatory biosynthesis, increased toward a CO_2_ vent core (Oppermann et al., 2010; Beulig et al., 2015). Furthermore, increasing abundances of putative anaerobes related to the *Chloroflexi* and *Firmicutes* phyla have been reported (Frerichs et al., 2013; Sáenz de Miera et al., 2014). Besides methanogens, ammonia oxidizing archaea related to the *Thaumarchaeota* were abundant at a CO_2_ vent core (Frerichs et al., 2013). Šibanc et al. (2014) reported oxygen concentration, soil pH and total nitrogen to be the strongest parameters shaping the archaeal community structure, while the bacterial composition is mainly shaped by the oxygen concentration of soil pore air. In addition to these studies on dry mofette soils, metagenomic analyses on deeply sourced CO_2_-saturated fluids from Crystal Geyser (Utah, USA) illuminated the potential influence of high CO_2_ concentrations on aquatic prokaryotic communities. This environment was dominated by populations capable of chemolithotrophy (“marine” and “freshwater” iron-oxidizing bacteria, sulfur-oxidizers and *Thiobacillus*-like Hydrogenophilales) and showed a broad diversity of uncultured species (Emerson et al., 2016). In the same system, Probst et al. (2017) observed autotrophic capabilities in organisms which account for over 70% of the community. Regarding microbial carbon fixation, high CO_2_ concentrations in the subsurface select for the Wood-Ljungdahl pathway and the Calvin-Benson-Bassham cycle, and for form II RuBisCOs, which are most likely adaptations to anaerobic and high CO_2_ conditions.

For the first time, this study presents a comprehensive insight into the microbial community structure of mineral and mofette waters influenced by active, strongly degassing CO_2_ conduits. Communities of aquatic bacteria and archaea were characterized using high-throughput sequencing of the 16S rRNA gene and quantitative PCR. To get indications for the origin of the waters in terms of depth and to check whether indications for microbial activity can be found, a chemical characterization of the waters using ICP-OES (cations), ion chromatography (anions) and isotope investigations using mass spectrometry (water phase: ^2^H_H2O_, ^18^O_H2O_ and ^34^S_SO4_, ^18^O_SO4_ and gas phase: methane, ethane and propane: ^2^H, ^13^C) were performed. Additionally, literature data of gas chemistry and isotope characteristics of the CO_2_-dominated gas (^13^C_CO2_, ^3^He/^4^He), based on multi-year investigations of the investigated sites, were used. With this interdisciplinary approach, we aimed (I) to retrieve a detailed geochemically characterization of the waters and the free, CO_2_ dominated gas phase, and to determine their origin, (II) to get a better understanding of the abundance and composition of aquatic microbial communities facing high CO_2_ partial pressures, (III) to identify key organisms and related metabolic pathways and (IV) to determine major community-shaping environmental factors.

## Geological background

The Cheb Basin (NW Bohemia, Czech Republic) is a shallow, neogene intracontinental basin that has formed since the Cenozoic at the intersection of the E-NE trending Eger Rift and the N-S trending Regensburg-Leipzig-Rostock seismoactive zone (Bankwitz et al., 2003; Fischer et al., 2014). The NW-trending pre-Neogene Mariánské Lázne Fault (MLF) forms the eastern boundary of the Cheb Basin and is marked by a 50–100 m high escarpment. The area is a non-volcanic region which features frequent earthquake swarms up to M_L_ 4.5 (hypocenter depths range between 6.5 and 11 km with some clusters down to 13 km; Fischer et al., 2014; Hainzl et al., 2016) and large-scale diffuse degassing of mantle-derived carbon dioxide at the surface that occurs in the form of CO_2_-rich mineral springs and wet and dry mofettes (Weinlich et al., 1998, 1999; Kämpf et al., 2013; Nickschick et al., 2015). Most of the earthquake activity (about 90% of the total seismic moment) is concentrated at the Nový Kostel focal zone (NKFZ), located at the intersection between the N-S trending Počatky-Plesná fault zone (PPZ) and the MLF (Fischer and Horálek, 2003). South of the Nový Kostel focal zone (NKFZ), the PPZ is characterized by intense CO_2_-degassing (mofette fields).

The migrating gas consists of up to >99 vol% CO_2_ and can contain traces of hydrogen, helium, argon, methane, oxygen or nitrogen (Bräuer et al., 2011, 2014). This migration of gas results in substantially changed soil gases and localized soil hypoxia (Kämpf et al., 2013; Nickschick et al., 2015). Carbon isotope signatures up to −70‰ of methane, a minor component of the CO_2_ dominated upstreaming gases, indicate an interaction between geological, geophysical and microbial driven processes in the deep subsurface (Bräuer et al., 2005, 2007). Mofettes share distinct geochemical features due to the exposure to elevated CO_2_ partial pressures. Acidification is a common feature which is reflected by low to very low pH values (3.5–4, Beaubien et al., 2008; 3.5–4.7, Rennert et al., 2011) and is negatively correlated to increasing CO_2_ concentration in soil gas or pore water (Mehlhorn et al., 2014; Rennert and Pfanz, 2016). Lowered pH and decreased redox potentials have been shown to increase metal mobilization in a mofette which could influence soil nutrient availability (Mehlhorn et al., 2014, 2016). Another common feature is the accumulation of organic matter (OM) in mofette soils (e.g., Beaubien et al., 2008; Beulig et al., 2015), which is a result of the assimilation of substantial quantities of geogenic CO_2_ via primary production by plants and subsurface carbon fixation (Oppermann et al., 2010; Beulig et al., 2016). A restricted degradation of OM by microorganisms under an increased CO_2_ atmosphere was indicated by the C/N ratio, the occurrence of undegraded plant material and high organic carbon contents (Rennert et al., 2011). Recently, Beulig et al. (2016) suspected that the permanent exclusion of meso-/macroscopic eukaryotes and related physiological capacities is the reason for the restricted degradation rather than an impaired biochemical potential of microorganisms.

## Methods

### Site description and sampling

**Figure 1:**
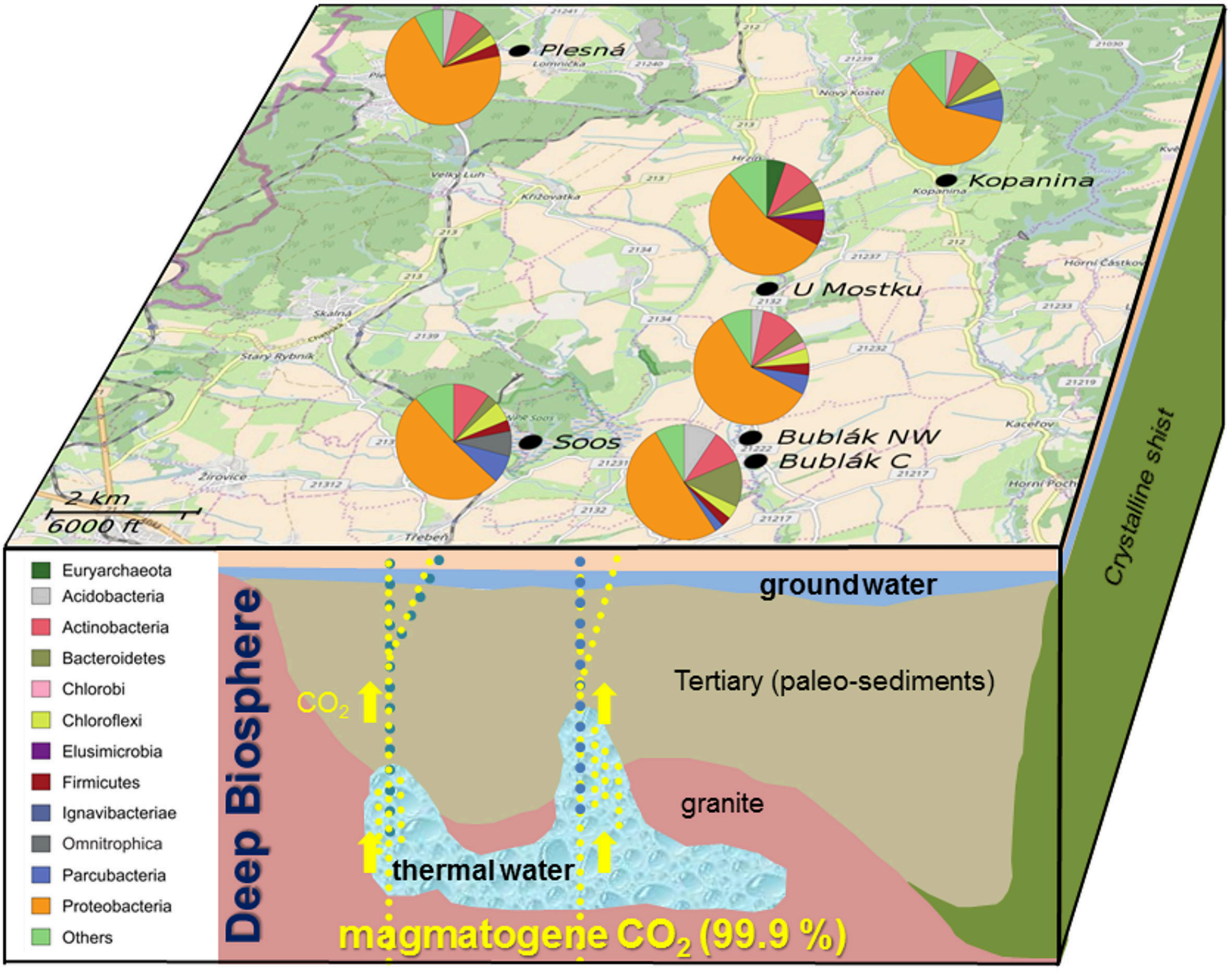
Pathway of magmatogene CO_2_, mixing with deep thermal water, paleo-sediment and ground water. Location of sampling sites and relative abundances of phyla determined by Illumina MiSeq sequencing of the 16S rRNA gene in different CO_2_ affected waters from the Cheb Basin, NW Bohemia. Only phyla with an abundance of at least 2% at a given site are shown. Map provided by © OpenStreetMap-Mitwirkende.

**Figure 2:**
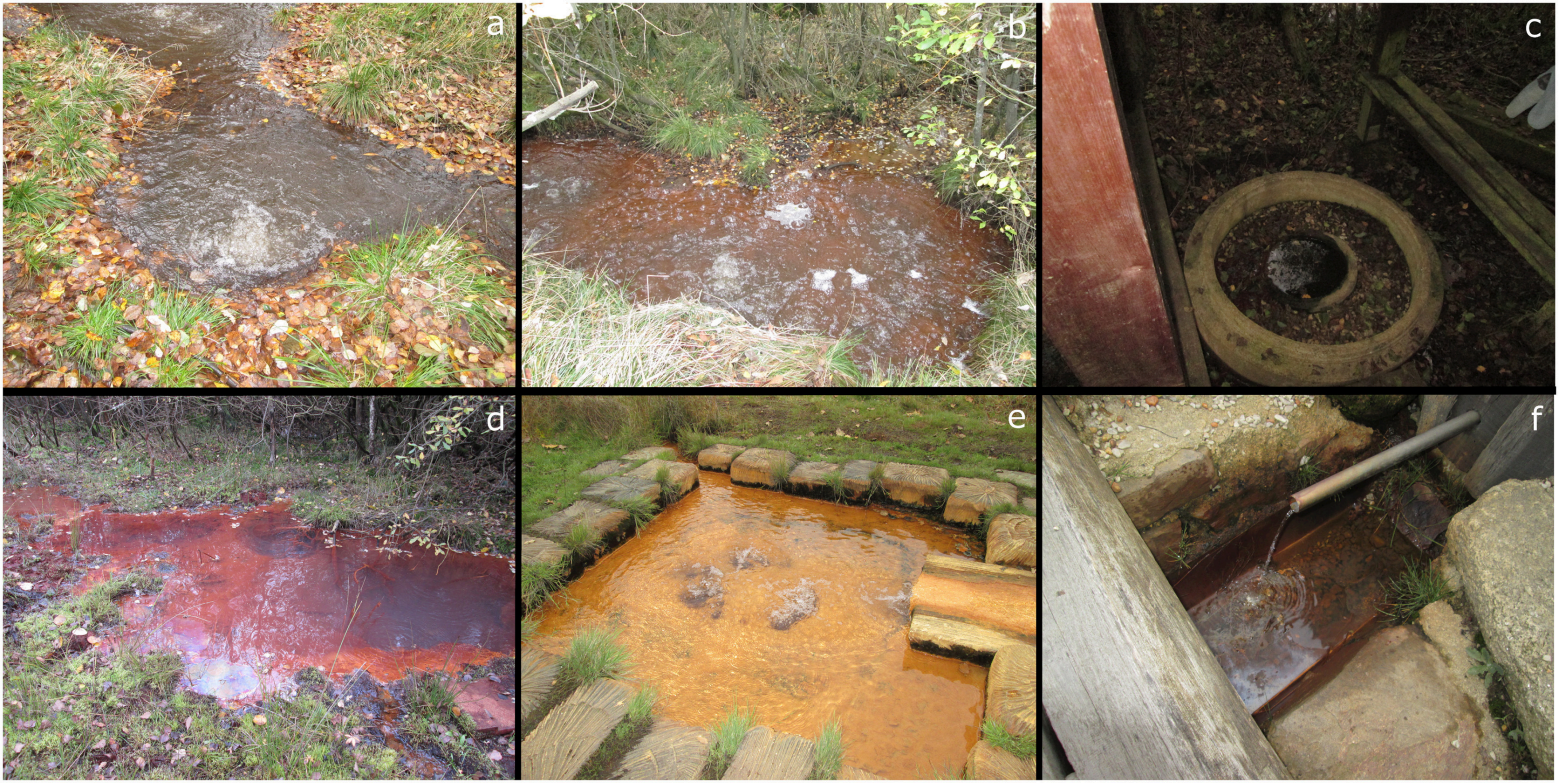
The six investigated sites. **(a)** Bublák C; **(b)** Bublák NW; **(c)** Kopanina; **(d)** Plesná; **(e)** Soos; **(f)** U Mostku.

All studied mofettes and mineral water springs are located in the Cheb Basin (Figures 1, 2). The sampling sites included two wet mofettes (“Bublák C” and “Bublák NW,” Figure 1 and Table 1), which were situated in swampy woodlands of the flood plain of the Plesná river. In contrast, the investigated mineral waters had a deeper origin, which is reflected in the respective hydrochemical properties. These included three waters, which had a shallow subsurface origin and were obtained from the Mostek u Križovatky pramen (“U Mostku”, Figure 1 and Table 1), a mineral water spring close to the Plesná river (“Plesná”, Figure 1 and Table 1) and a freshwater spring (“Kopanina”, Figure 1 and Table 1) close to a brook. The fourth water has a deeper origin and was obtained from the Císarský pramen at the Soos area (“Soos”, Figure 1 and Table 1). Sampling (water and gas) took place in March, April, October and December 2014 to capture a possible seasonality. The samples collected in October 2014 (at 29.10.2014) were used for detailed microbial studies. For microbiological investigations at each site, a 5 L amber stained laboratory glass bottle was filled with water either from a pond or directly from a wellhead. To show the distinctness of the aquatic microbial communities of CO_2_-influenced waters, related soils need to be investigated. Thereby, the top five centimeters directly next to a wet mofette (Bublák C), a mineral water spring (Plesná) and sediment from the Císarský pramen well (Soos) were sampled. Additionally, several smaller volumes of water were sampled for the analysis of element hydrochemistry and isotope (^18^O, ^2^H, ^34^S) hydrochemistry. Depending on filter clogging, varying volumes (2.5–5.5 l) of water were filtered in replicate using 0.2 μm cellulose-acetate filters (Sartorius AG, Germany) and stored at −20°C till DNA extraction. The gas was sampled using glass vessels with two stopcocks for isotope analysis of methane (^13^C, ^2^H), ethane and propane (^13^C). The vessels were filled with spring water, which was replaced by the free gas bubbling out of the water in the glass vessel.

**Table 1:** Water/gas chemistry and related isotopic analysis data of mofette and mineral waters from the Cheb Basin, NW Bohemia.

**Parameter [unit]**	**Sample site**					
	**Soos spring^e^**	**Plesná spring**	**U Mostku spring**	**Bublák C mofette**	**Bublák NW mofette**	**Kopanina spring**
Latitude [°N]^f^	50.148	50.226	50.174	50.143	50.144	50.206
Longitude [°E]^f^	12.403	12.370	12.444	12.454	12.450	12.458
Temp. [°C]^h^	17.4	7.9	9.2	8.9	7.9	8.7
Cond. [μS cm^−1^]^h^	6,700	249	431	121	253	248
O_2_ [%]^h^	1.5	38.7	19.4	13.8	6.3	6.5
O_2_ [mg L^−1^]^h^	0.10	4.34	2.12	1.53	0.72	0.72
pH^h^	6.0	5.1	5.4	4.7	4.8	4.2
TOC [mg L^−1^]^h^	3.0	3.7	4.5	6.2	18.0	5.1
DOC [mg L^−1^]^h^	2.9	3.0	4.4	5.0	7.9	4.6
CO_2_ dissolved [mg L^−1^]	n.a.	1,800^c^	1,855^c^	2,043^c^	n.a.	1,912^c^
${\text{HCO}}_{3}^{-}$	n.a.	n.a.	n.a.	n.a.	n.a.	n.a.
Gas flow [L h^−1^]	n.a.	~144^b^	~1^c^	~19,112^d^	~23,988^d^	~1^c^
**GAS COMPOSITION**						
CO_2_ [vol.%]	99.99^a^	99.55^b^	85.97^c^	99.56^c^	99.19	80.42^c^
N_2_ [vol.%]	0.01^a^	0.31^b^	12.95^c^	0.32^c^	0.78	18.54^c^
Ar [ppmv]	n. a.^a^	83^b^	3,300^c^	80^c^	15	2,400^c^
O_2_ [ppmv]	n. a.^a^	1,330^b^	7,300^c^	120^c^	191	7,300^c^
CH_4_ [ppmv]	2.0^a^	15.0^b^	199.0^c^	2.5^c^	3.1	64.1^c^
He [ppmv]	0.9^a^	0.7^b^	75.8^c^	18.7^c^	44.1	529.0^c^
**Isotope ratios**						
δ^13^C_CO2_ [‰]	−3.60^a^	−3.04^b^	−0.83^c^	−1.98^c^	−1.86^d^	−1.75^c^
δ^13^C_CH4_ [‰]^g^	−56.2	−52.9	n.a.	−53.1	−51.3	n.a.
δ^2^H_CH4_ [‰]^g^	−204	−198	n.a.	−209	−196	n.a.
δ^13^C_C2H6_ [‰]^g^	−31.3	n.a.	n.a.	−33.7	−33.1	n.a.
δ^13^C_C3H8_ [‰]^g^	−27.6	n.a.	n.a.	−29.5	−29.9	n.a.
^3^He/^4^He [R_a_]	3.43^a^	2.80^b^	5.56^c^	5.89^c^	5.66^d^	4.60^c^
**WATER COMPOSITION^h^**						
**Cations [mg L^−1^]**						
K	41.0	3.4	88.0	2.5	5.8	4.8
Mg	22.0	5.3	2.5	3.1	7.2	5.6
Na	1,554.0	10.0	31.2	7.7	7.2	6.0
Ca	68.0	7.5	23.0	11.0	24.0	21.0
Fe^2^^+^	37.0	33.0	1.7	0.31	4.0	1.3
Mn	1.6	0.74	<0.1	<0.1	0.28	0.28
**Anions [mg L^−1^]**						
F	2.2	0.1	<0.1	0.1	0.1	0.4
Formic acid	n.a.	<0.1	<0.1	<0.1	<0.1	n.a.
Acetic acid	n.a.	n.a.	1.6	n.a.	n.a.	n.a.
Propionic acid	n.a.	n.a.	<0.1	n.a.	n.a.	n.a.
Cl	594.7	4.6	1.9	6.7	8.5	10.2
Br	1.3	n.a.	n.a.	n.a.	n.a.	n.a.
NO_2_	n.a.	<0.1	n.a.	n.a.	<0.1	<0.1
NO_3_	2.3	<0.1	n.a.	0.1	n.a.	0.7
SO_4_	1,807.0	14.4	2.9	6.4	45.1	68.4
**Isotope ratios [‰]^i^**						
δ^18^O_H2O_	−10.5	−10.1	−8.9	−9.7	−8.8	n.a.
δ^2^H_H2O_	−67.5	−69.0	−63.8	−63.5	−62.0	n.a.
δ^34^S_SO4_	5.6	0.7	4.7	0.5	15.2	n.a.
δ^18^O_SO4_	4.0	2.1	2.5	7.1	13.3	n.a.

+ n.a. not analyzed;

a Kämpf et al. (2007);

b Bräuer et al. (2008);

c Bräuer et al. (2011);

d Kämpf et al. (2013);

e Cisarsky pramen;

f coordinates: UTM WGS 84, Zone 33;

g sampling date: 02.12.2014;

h measurement and sampling date: 29.10.2014;

i *sampling date: 10.04.2014*.

### Geochemical and isotope analysis of waters

Electrical conductivity (EC), pH, O_2_ and water temperature were directly measured in the field (Multi 3420 digital analyzer, WTW GmbH Weilheim). To investigate the main cations (K^+^, Mg^2+^, Na^+^, Ca^2+^, Fe^2+^, and Mn^2+^), water samples were filtered (<0.2 μm) and acidified to pH <2 with HNO_3_ for storage in 50 ml PP bottles at 4°C. Analysis took place at the GFZ German Research Centre for Geosciences in Potsdam, Germany by ICP-OES (VarianVista-MPX). Standards (plasma standard solution, Johnson Matthey Company), containing all of the investigated compounds, were measured in different concentrations every day.

The standard deviation of sample and standard quantification is <2% for Mg^2+^, Ca^2+^, Fe^2+^, and Mn^2+^, <3% for Na^+^ and <4% for K^+^ and was determined by at least three measurements. For the investigation of anions, the water samples were filled in 1 L glass bottles in the field. The samples were analyzed at the GFZ German Research Centre for Geosciences by ion chromatography (IC) with conductivity detection (ICS 3000, Dionex) to determine the content of F^−^, ${\text{PO}}_{4}^{3-}$, NO^2−^, NO^3−^, Br^−^, Cl^−^, and ${\text{SO}}_{4}^{2-}$ and organic acids (formate, acetate, propionate). Standards containing all of the investigated compounds were measured in different concentrations every day. The standard deviation of sample and standard quantification is below 10% (determined by at least two measurements).

For sulfate isotope analyses, dissolved sulfate stored in 0.5 up to 1 L PP bottles (depending on the quantity of dissolved sulfate) was precipitated using BaCl_2_·2H_2_O. The precipitated BaSO_4_ was collected by filtration through nitrocellulose membranes, washed to remove residual BaCl_2_ and dried at 50°C. Sulfur isotopic compositions were measured after conversion of BaSO_4_ to SO_2_ using an elemental analyzer (continuous flow flash combustion technique) coupled with an isotope ratio mass spectrometer (Delta S, ThermoFinnigan, Bremen, Germany) at the stable isotope laboratory of the Helmholtz Centre for Environmental Research–UFZ, Germany.

Analytical errors of the measurement of more than ±0.3‰ and results are reported in delta notation (δ^34^S) as part per thousand (‰) deviation relative to the Vienna Cañon Diablo Troilite (VCDT) standard. Oxygen isotope analysis of sulfate was performed using a delta plus XL mass spectrometer (ThermoFinnigan, Bremen, Germany) with an analytical precision of more than ±0.5‰. Results of oxygen isotope measurements are expressed in delta notation (δ^18^O_SO4_) as part per thousand (‰) deviation relative to Vienna Standard Mean Ocean Water (VSMOW). For normalization of the δ^34^S and δ^18^O_SO4_ data, the IAEA-distributed reference material NBS 127 (BaSO_4_) was used. The assigned values were +20.3‰ (VCDT) δ^34^S and +8.6‰ (VSMOW) for δ^18^O_SO4_.

Measurements of stable isotopes of δ^18^O and δ^2^H in the water samples were performed at the stable isotope laboratory of the UFZ in Halle/Saale, Germany using a laser-based analyzer (L1102-I, Picarro Inc.). This instrument has an analytical precision of 0.5‰ for δ^2^H and 0.1‰ for δ^18^O. For normalization of the δ^18^O water data, the IAEA-distributed reference materials VSMOW and SLAP were used.

### Isotope analysis of gases

The carbon isotope ratios of CO_2_, CH_4_, and higher gaseous hydrocarbons and hydrogen isotope ratios of CH_4_ were measured at the stable isotope laboratory of Hydroisotop GmbH Schweitenkirchen using a GC-IRMS equipped with a purge and trap device. The line consists of PTA−3000 Purge and Trap autosampler (IMT Germany), a Trace GC Ultra gas chromatograph (Thermo Scientific) with Hayesep Q separation column (VICI) and helium as carrier gas, and Delta V isotope ratio mass spectrometer–IRMS (Thermo Scientific). The Isodat 3 software was used to evaluate the signals. Results are reported in δ values relative to International standards: V-PDB for carbon and V-SMOW for hydrogen. The instrumental error of δ^13^C in CO_2_, CH_4_ and higher hydrocarbons is ±0.5‰ and that of δ^2^H is ±5‰.

### Nucleic acids extraction

Genomic DNA of the sampled waters was extracted from filters using the PowerWater® DNA Isolation Kit (MO BIO Laboratories Inc., USA) according to the manufacturer's specifications with minor changes to the protocol. Once 100 μl of elution buffer was added, the samples were incubated at 55°C for 5 min before the final centrifugation step. The extracted DNA was stored at −20°C.

Genomic DNA from 0.5 mg of the sampled sediments was extracted using the PowerSoil® DNA Isolation Kit (MO BIO Laboratories Inc., USA).

These DNA preparations were used as a template for the quantification by quantitative PCR (qPCR) and next-generation sequencing.

### Quantification of bacterial 16S rRNA genes and functional genes

Quantitative polymerase chain reaction was used to quantify total bacterial abundances and the functional genes of sulfate reducing bacteria and methanogenic archaea. All qPCR essays were performed in triplicates on a CFX96 Real-time thermal cycler (Bio-Rad Laboratories Inc., USA) and contained 12.5 μl iTaq™ Universal SYBR® Green Supermix (ThermoFisher Scientific Inc., USA), 8.5–10.5 μl PCR water, each 0.5 μl of forward and reverse primer (20 μM) and 1–3 μl template. The setup was optimized for each target regarding the cycler program, used volumes and dilution factors of the samples. The quantification of the bacterial 16S rRNA gene was based on the primers 331F (5′-TCCTACGGGAGGCAG-CAGT-3′) and 797R (5′-GGACTACCAGGGTATCTAATCCTGTT-3′) (Nadkarni et al., 2002). After an initial denaturing phase of 5 min at 98°C, the cycler included 40 cycles of 5 s at 98°C, 20 s at 57°C and 60 s at 72°C plus the plate read. The quantification of sulfate reducers was based on the primers dsr2060F (5′-CAACATCGTYCAYACCCAGGG-3′) (Geets et al., 2006) and dsr4R (5′-GTGTAGCAGTTACCGCA-3′) (Wagner et al., 1998) targeting the dissimilatory sulfite reductase β-subunit (*dsrB*) gene and included an initial denaturing for 10 min at 95°C, followed by 40 cycles of 30 s at 95°C, 60 s at 60°C, 60 s at 72°C and 3 s at 80°C plus the plate read. The quantification of methanogenic archaea was based on the primers mlas-F (5′-GGTGGTGTMGGDTTCACMCARTA-3′) and mcrA-R (5′-CGTTCATBGCGTAGTTVGGRTAGT-3′) (Steinberg and Regan, 2009) targeting the methyl coenzyme M reductase (*mcrA*) gene and included an initial denaturing for 3 min at 95°C, followed by 40 cycles of 5 s at 95°C, 20 s at 58.5°C, 30 s at 72°C and 3 s at 80°C plus the plate read. All cycling programs included a melting curve from 60 to 95°C with 0.5°C steps per plate read. The analysis of quantification data was performed with the CFX Manager™ Software (Bio-Rad Laboratories Inc., USA).

### Illumina MiSeq sequencing

Unique combinations of tagged 515F (5′-GTGCCAGCMGCCGCGGTAA-3′) and 806R (5′-GGACTACHVGGGTWTCTAAT-3′) (Caporaso et al., 2011) primers were assigned to each sample. The samples were processed in duplicates and pooled afterwards to reduce PCR variability. Additionally, technical replicates for the sequencing of each sample were produced to reduce sequencing variability. The PCR was performed on a T100™ Thermal Cycler (Bio-Rad Laboratories Inc., USA) in 25 μl reactions, containing 12.5 μl iTaq™ Universal SYBR® Green Supermix (ThermoFisher Scientific Inc., USA), 8.75 μl PCR water, each 0.625 μl of forward and reverse primer (20 μM) and 2.5 μl genomic DNA using following cycler program: Initial denaturing step for 3 min at 95°C followed by 10 cycles of 1 min at 94°C, 1 min at 53°C (−0.2°C/cycle) and 1 min at 72°C, followed by 20 cycles of 1 min at 94°C, 1 min at 50°C and 1 min at 72°C, followed by a final extension step for 10 min at 72°C. All samples were pooled by adding an equal amount of DNA (60 ng DNA per sample). Subsequently, a purification of the PCR product pool was achieved by using the Hi Yield Gel/PCR DNA Fragment Extraction. The Illumina MiSeq sequencing was performed by EuroFins Scientific SE, Luxembourg.

### Bioinformatics and statistical analysis

Sequencing was performed on an Illumina MiSeq (2 × 250 bp). Reads were demultiplexed using CutAdapt (options: e0.1; trim-n; Martin, 2011). Read pairs were merged using PEAR (options: Q25; p10^−4^; o20; Zhang et al., 2014). QIIME (version 1.9.1) (Caporaso et al., 2010) was employed for microbiome analysis. USEARCH (Edgar, 2010) was used for the detection and removal of chimeric sequences. The SILVA database (version 128) (DeSantis et al., 2006) was utilized for open-reference OTU clustering (97% sequence similarity) and taxonomic assignments. Singletons and OTUs assigned to chloroplasts were removed. For the processing and visualization of the obtained OTU table, R, CANOCO 5 (Šmilauer and Lepš, 2014) and PAST3 (Hammer et al., 2001) software was used. For alpha diversity analyses, the data were rarefied to 61,042 reads per sample. Alpha diversity was estimated using the Shannon's H index, Faith's Phylogenetic diversity and Pielou's evenness. Beta diversity was calculated using weighted UniFrac distance. Similarity Percentages (SIMPER) analyses were used to determine the main drivers of dissimilarity of community composition. Sequencing data were submitted to the European Nucleotide Archive (http://www.ebi.ac.uk/ena) under accession number PRJEB20063.

## Results

### Characterization of mofette and mineral waters

Distinct differences in hydrochemical properties were measured in water samples from different locations (Table 1). The waters were acidic with pH values ranging from 4.2 to 6.0 and showed low contents of dissolved oxygen of 0.10–4.34 mg L^−1^. The deep thermal water (Soos) differed from all other waters featuring a relative high temperature of 17.4°C and conductivity of 6,700 μS cm^−1^, indicating its deeper subsurface origin. Additionally the concentration of several ions, for instance magnesium, calcium, sodium, manganese, chloride and sulfate, was very high compared to the other waters. The mineral water springs (Soos, Plesná) showed especially high concentrations of Fe^2+^ at 37 and 33 mg L^−1^ respectively. The total organic carbon (TOC) and dissolved organic carbon (DOC) concentrations varied greatly: Lowest TOC values could be observed in the mineral water springs (Soos: 3.0 mg L^−1^, Plesná: 3.7 mg L^−1^) and highest in the mofette waters (Bublák C: 6.2 mg L^−1^, Bublák NW: 18.0 mg L^−1^). A similar trend was detected regarding the DOC concentrations. Mineral water springs showed lowest (Soos: 2.9 mg L^−1^, Plesná: 3.0 mg L^−1^) and mofette waters highest (Bublák C: 5.0 mg L^−1^, Bublák NW: 7.9 mg L^−1^) amounts of DOC. Furthermore, only the shallow subsurface water (U Mostku) showed detectable amounts of acetic acid (1.6 mg L^−1^). The concentrations of other organic acids (e.g., formic acid, propionic acid) were under the detection limit. Nitrite was not detectable. The nitrate concentration was overall low, and the deep mineral water (Soos) showed the highest concentration with 2.3 mg L^−1^.

The δ^2^H vs. δ^18^O values of sampled waters showed similar values (δ^2^H from −69 to −62.0‰ SMOW and δ^18^O from −8.8 to −10.5‰ SMOW). The δ^18^O vs. δ^34^S values of the dissolved sulfate of the waters showed large differences in the values (δ^18^O from 2.1 to 13.3‰ and δ^34^S from 0.5 to 15.2‰). In most cases, the sulfate content amounts to <100 mg L^−1^, except for Soos (1,807.0 mg L^−1^).

### Chemical and isotopic composition of free gas samples

The chemical composition (in % by vol.) and the isotopic composition (^3^He/^4^He ratio as R/R_a_, R_a_: atmospheric ^3^He/^4^He ratio and δ^13^C_CO2_ in ‰-V-PDB) of gas samples collected from the gas of bubbling waters are reported in Table 1. The gas compositions of the mineral springs Soos, Plesná and both Bublák mofettes were dominated by CO_2_ (>99%), while the springs U Mostku and Kopanina showed lower CO_2_ concentrations (<90%).

The δ^13^C_CO2_ values ranged between −3.6‰ and −0.83‰ relative to the V-PDB standard (Kämpf et al., 2007, 2013; Bräuer et al., 2008, 2011). The highest ^3^He/^4^He ratios were obtained along the PPZ (Bublák C: 5.89 R_a_, Bublák NW: 5.66 R_a_ and U Mostku: 5.56 Ra), whereas at Kopanina, located at the MLF the ^3^He/^4^He ratio is lower (4.6 R_a_). The lower portions of mantle-derived helium were shown outside of these fault zones at the Soos mineral spring (3.43 R_a_) and the lowest on the periphery of the degassing center of the Cheb Basin at the Plesná mineral spring (2.8 R_a_). The δ^13^C_CH4_ values ranged between −56.2 and −51.3‰ relative to the V-PDB standard and the δ^2^H_CH4_ values ranged between −209 and −196‰ relative to the V-SMOW standard.

### Abundances of microorganisms

Clear differences in 16S rRNA gene abundances were detected between waters from surface and deep locations (Table 2). Abundances ranged from 8.8 × 10^5^ copies L^−1^ (Soos) to 1.7 × 10^10^ copies L^−1^ (Bublák NW), while in most cases values between 10^8^ and 10^9^ copies L^−1^ could be observed. The *dsrB* gene was successfully quantified in each water except for the deep mineral water (Soos). The abundance of this gene ranged between 1.9 × 10^5^ copies L^−1^ (Plesná) and 2.5 × 10^7^ copies L^−1^ (Bublák NW), following a similar trend as the 16S rRNA gene abundances. The quantification of the *mcrA* gene only succeeded for both Bublák mofettes (Bublák C; Bublák NW) and the shallow groundwater (U Mostku). The abundances were comparatively low with 6.0 × 10^5^ genes L^−1^ (Bublák NW), 1.6 × 10^5^ genes L^−1^ (Bublák C) and 8.8 × 10^4^ (U Mostku). *McrA* gene abundances of the remaining waters were under the limit of detection (~10^4^ genes L^−1^).

**Table 2:** Abundances of bacterial 16S rRNA, *dsrB* and *mcrA* genes revealed by quantitative PCR and calculated alpha diversity values based on the OTUs in the investigated CO_2_ affected mofette and mineral waters from the Cheb Basin, NW Bohemia.

**Parameter [unit]**	**Sample site**					
	**Soos spring**	**Plesná spring**	**U Mostku spring**	**Bublák C mofette**	**Bublák NW mofette**	**Kopanina spring**
**QUANTIFICATION**						
Bacterial 16S rRNA [gene copies L^−1^]	8.8 × 10^5^ ± 1.2 × 10^5^	2.0 × 10^9^ ± 1.5 × 10^8^	3.7 × 10^8^ ± 5.0 × 10^7^	7.8 × 10^9^ ± 6.0 × 10^8^	1.7 × 10^10^ ± 5.4 × 10^7^	3.8 × 10^8^ ± 2.8 × 10^7^
*dsrB* [gene copies L^−1^]	Too low	1.9 × 10^5^ ± 2.1 × 10^4^	2.1 × 10^6^ ± 7.1 × 10^4^	3.4 × 10^6^ ± 3.0 × 10^5^	2.5 × 10^7^ ± 1.7 × 10^6^	8.9 × 10^5^ ± 6.4 × 10^4^
*mcrA* [gene copies L^−1^]	Too low	Too low	8.8 × 10^4^ ± 9.0 × 10^3^	1.6 × 10^5^ ± 4.9 × 10^3^	6.0 × 10^5^ ± 4.1 × 10^4^	Too low
**ALPHA DIVERSITY**						
Shannon's H	7.2 ± 0.43	6.1 ± 0.32	7.2 ± 0.06	8.6 ± 0.01	8.8 ± 0.06	8.1 ± 0.06
Phylogenetic diversity	154 ± 20	161 ± 8	166 ± 2.7	211 ± 2	269 ± 1	207 ± 4
Evenness	0.62	0.51	0.69	0.69	0.60	0.65

*Gene abundance values represent mean values from triplicates. Before calculation of alpha diversity indices, each sample was rarefied 10 times to 61,042 sequences. Mean values of the iterations and related replicates are shown*.

### Diversity and microbial community composition

In total, 1.63 million reads were obtained after merging the forward and reverse reads and demultiplexing. After quality filtering and deletion of chimeric sequences, 1.46 million high quality reads remained in the sample set. The amount of reads per sample ranged from 68,784 to 477,069 with a mean value of 129,181 (Table S1). Hereby, rarefaction analyses showed that no sample exhibited a conspicuous increase of its Shannon's H index after including more than 10,182 sequences (Figure S1, Table S2). All samples have been sufficiently covered by sequencing, since an increasing number of reads per sample does not bias diversity. As indicated by boxplots analyses (Figure S2), the replicates of a sample showed only a small distance to each other, whereby larger distances were observed between different sites. A total of 25,909 OTUs were calculated. Before analysis, we removed chloroplast-related OTUs (3.11% of total read counts) and OTUs that could not be assigned to any domain (5.55% of total read counts) from the data set. After taxonomic classification, 1,399 putative genera were obtained.

The alpha diversity of the samples was calculated using Shannon's H index (Table 2). The alpha diversity of the water obtained from a surface mineral spring (Plesná) showed the lowest microbial diversity, followed by both subsurface waters (Soos; U Mostku). Generally the diversity of the surface waters (Bublák C, Bublák NW, and Kopanina) was higher. The differences between surface and subsurface waters are similarly observable in the Faith's PD index (Table 2). None of the considered environmental parameters showed a significant correlation with the calculated alpha diversity indices.

**Figure 3:**
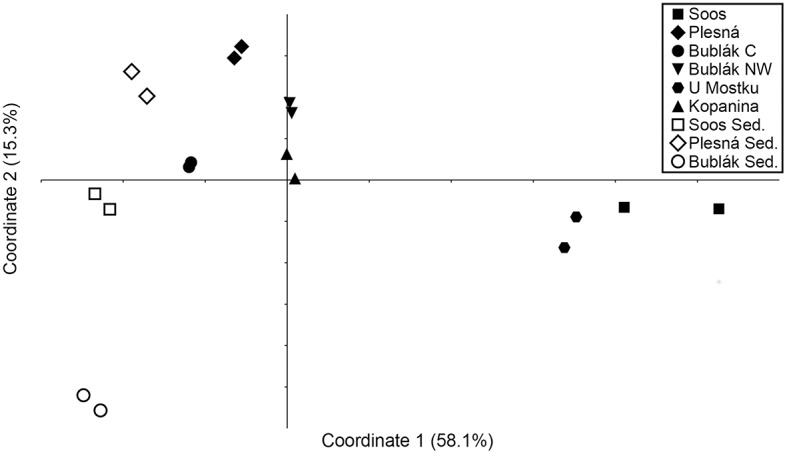
Principal coordinates analysis (PCoA) plot derived from weighted UniFrac distance between the investigated microbial communities. Axis 1 and Axis 2 explain 73.4% of the variance.

**Figure 4:**
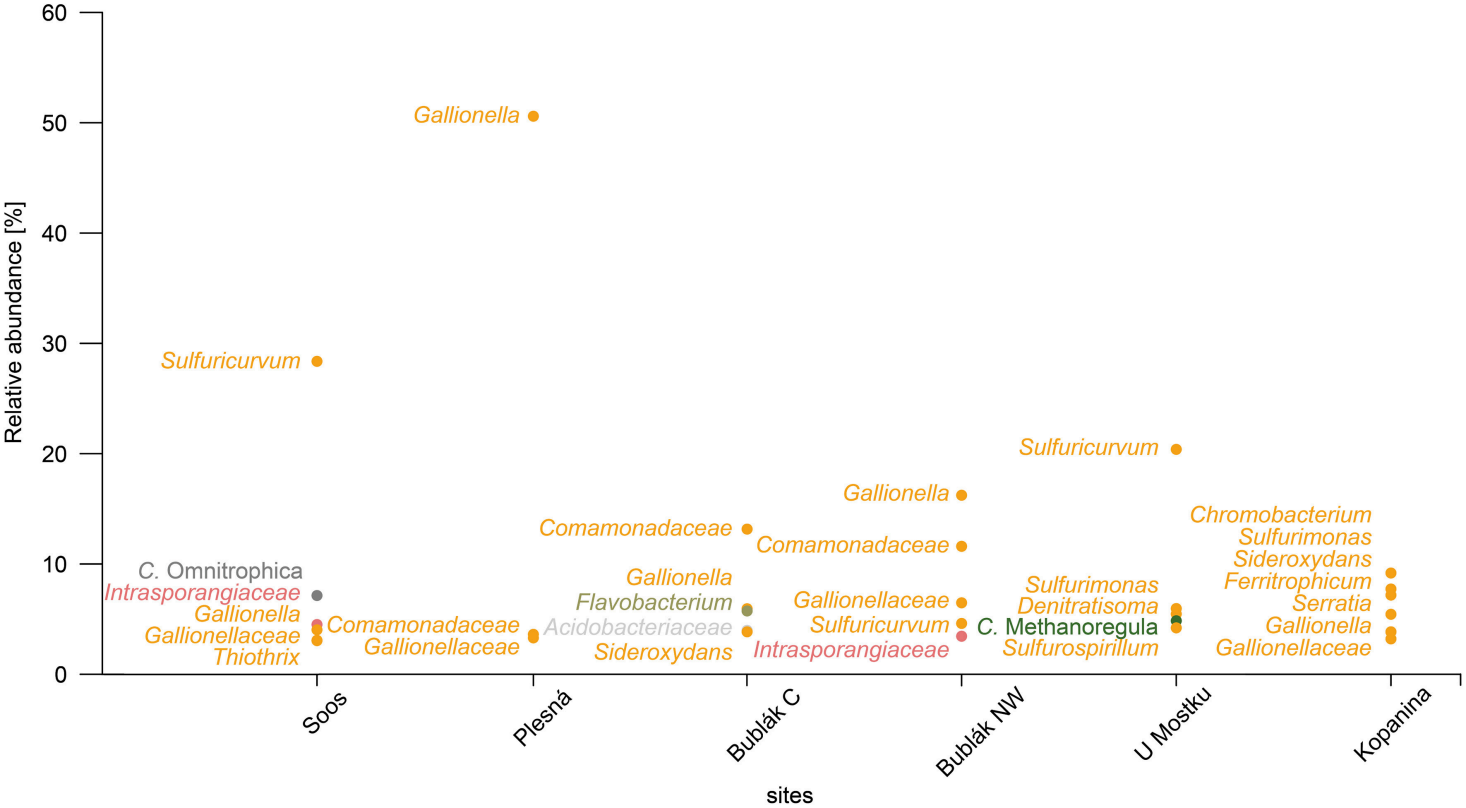
Dominant groups at the lowest assignable taxonomic level in the microbial communities of CO_2_ affected waters. Labels are shown only for groups with average abundances over 3%. Groups of taxa belonging to the same phylum are colored according to Figure 1.

To access beta diversity and therefore the differences in microbial community composition in the mofette and mineral waters, weighted UniFrac distance was calculated. The clustering of the samples in the corresponding PCoA (Figure 3) showed that the microbial communities found in subsurface waters (U Mostku, Soos) differed from the surface waters (Plesná, Bublák C, Bublák NW, and Kopanina). SIMPER test revealed *Sulfuricurvum, Gallionella, Comamonadaceae, Omnitrophica, Denitratisoma*, and *Sulfurimonas* to be the main drivers of dissimilarity (>1.5% contribution) between surface and subsurface waters (Table S3). Microbial communities of related sediments differed from the respective waters. Abundances of different phylotypes in CO_2_ affected waters were analyzed on phylum and, depending on the possibility of an assignment, on the lowest identifiable taxonomic level. Generally, the phylum *Proteobacteria* was very abundant across all mofette and mineral waters (Figure 1). However, the abundances of *Proteobacteria*-related families and genera and other phyla differed substantially (Figures 1, 4, Table S4). In the following part, the most abundant groups in the investigated waters are presented.

At the phylum level, the microbial community of the deep mineral waters in Soos was composed of *Proteobacteria* (51.6%), *Actinobacteria* (10.29%), *Parcubacteria* (7.84%), *Omnitrophica* (7.15%), and *Chloroflexi* (5.61%). In comparison to the other investigated waters, a large fraction of unassignable sequences was found. Most abundant genera belonged to the phylum *Proteobacteria*, namely *Sulfuricurvum* (28.37%), *Gallionella* (4.03%), *Thiothrix* (3.05%), and *Sulfurimonas* (2.86%). A similar profile was observed for the shallow groundwater (U Mostku): *Proteobacteria* (55.95%), *Actinobacteria* (9.02%), *Firmicutes* (7.09%), and *Bacteroidetes* (5.96%) represented the most abundant phyla, while the most abundant genera consisted of *Sulfuricurvum* (20.39%), *Sulfurimonas* (5.96%), *Denitratisoma* (5.47%), and *Sulfurospirillum* (4.21%). Unique for this water was the occurrence of a larger fraction related to archaeal taxa, namely the phylum *Euryarchaeota* (5.29%) and the corresponding genus *Methanoregula* (4.85%). In contrast to these two waters, microbial communities of the remaining CO_2_ affected waters showed less OTUs related to the sulfur cycling (e.g., *Sulfuricurvum* or *Sulfurimonas)*. The community of the mineral spring water (Plesná) was dominated by *Proteobacteria* (70.08%) and particularly by *Gallionella*-related OTUs (50.59%). OTUs related to *Sideroxydans* (1.35%) and *Bacteriovorax* (1.67%) were significantly less abundant. The communities of the mofette waters (Bublák C; Bublák NW) shared several features in terms of abundant phylotypes. Besides, a large portion of *Proteobacteria*-related OTUs (50.9%; 58.53%), smaller abundances of *Bacteroidetes* (13.27%; 3.8%), *Acidobacteria* (9.38%; 3.4%) and *Actinobacteria* (9.25%; 10.49%) were present in both habitats. Similar to some other waters, smaller fractions of *Gallionella*-related OTUs (5.94%; 16.23%), *Sideroxydans* (3.85%; 1.78%), *Sulfuricurvum* (1.33%; 4.61%) and *Sulfurimonas* (2.47%; 1.04%) were observed. The occurrence of greater fractions of *Flavobacteriaceae* and especially OTUs related to *Flavobacterium* (5.52%) was a unique feature of the mofette water at Bublák C. On a phylum level, the community structure of the surface water (Kopanina) was similar to the other surface waters, with high abundances of *Proteobacteria* (60.28%), *Bacteroidetes* (6.84%), *Actinobacteria* (6.57%), and *Parcubacteria* (6.71%). However, on a deeper taxonomic level, several differences compared to all other waters were observed. The most abundant genera were *Chromobacterium* (9.18%), *Sulfurimonas* (7.73%), *Sideroxydans* (7.18%), and *Ferritrophicum* (5.44%).

**Figure 5:**
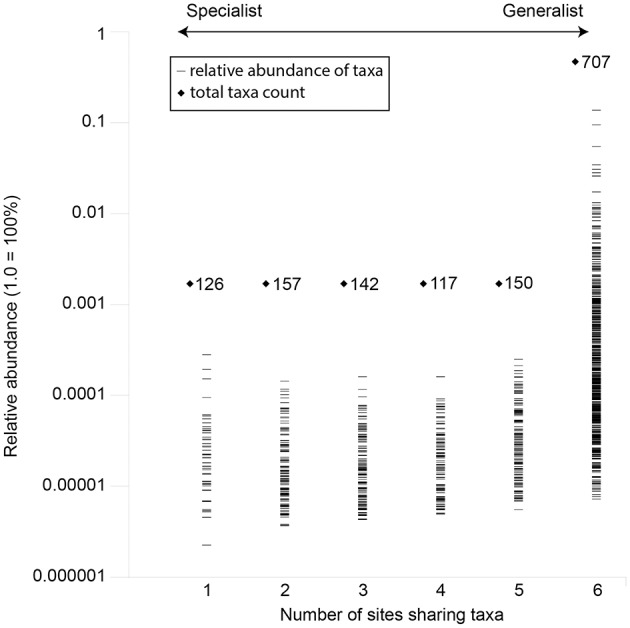
Occurrence and mean abundance of generalists and site-specific specialists across the investigated mofette sites. Unassigned taxa were not considered.

Overall, the investigated waters shared 707 out of 1,399 assignable genera. This shared fraction (generalists) comprised 50.5% of all taxa and 98.9% of the total read counts in the data set (Figure 5). On the other hand, unique and site specific genera (specialists) were rare and made up 126 assignable genera (9% of all taxa) and 0.05% of the total read counts. Based on the total read counts in the data set, most abundant common taxa were the iron oxidizers *Gallionella* (13.71%), another *Gallionellaceae*-related taxon (3.06%), *Sideroxydans* (2.59%), the sulfur oxidizers *Sulfuricurvum* (9.46%) and *Sulfurimonas* (3.43%) and taxa related to *Comamonadaceae* (5.47%) and *Intrasporangiaceae* (2.81%). None “specialist” taxa showed relative abundances above 0.03%.

In all investigated waters more than 50 obligate marine taxa, such as Nitrospinaceae, Maritimimonas, Oceanobacillus, Marinobacter, Marinilabiaceae, Pseudohongiella, Acidimicrobiaceae (marine group), Desulfatiferula, Dehalococcoidia (GIF3, GIF9, MSBL5, Napoli-4B) and Thaumarchaeota (marine group) were identified (Table S4). The deep mineral waters of Soos showed the largest fraction of these marine taxa (rel. abundance 2.22%), whereby the relative abundance at the other sites was smaller (0.09–0.28%).

### Correlation between environmental parameters and the microbial communities

**Figure 6:**
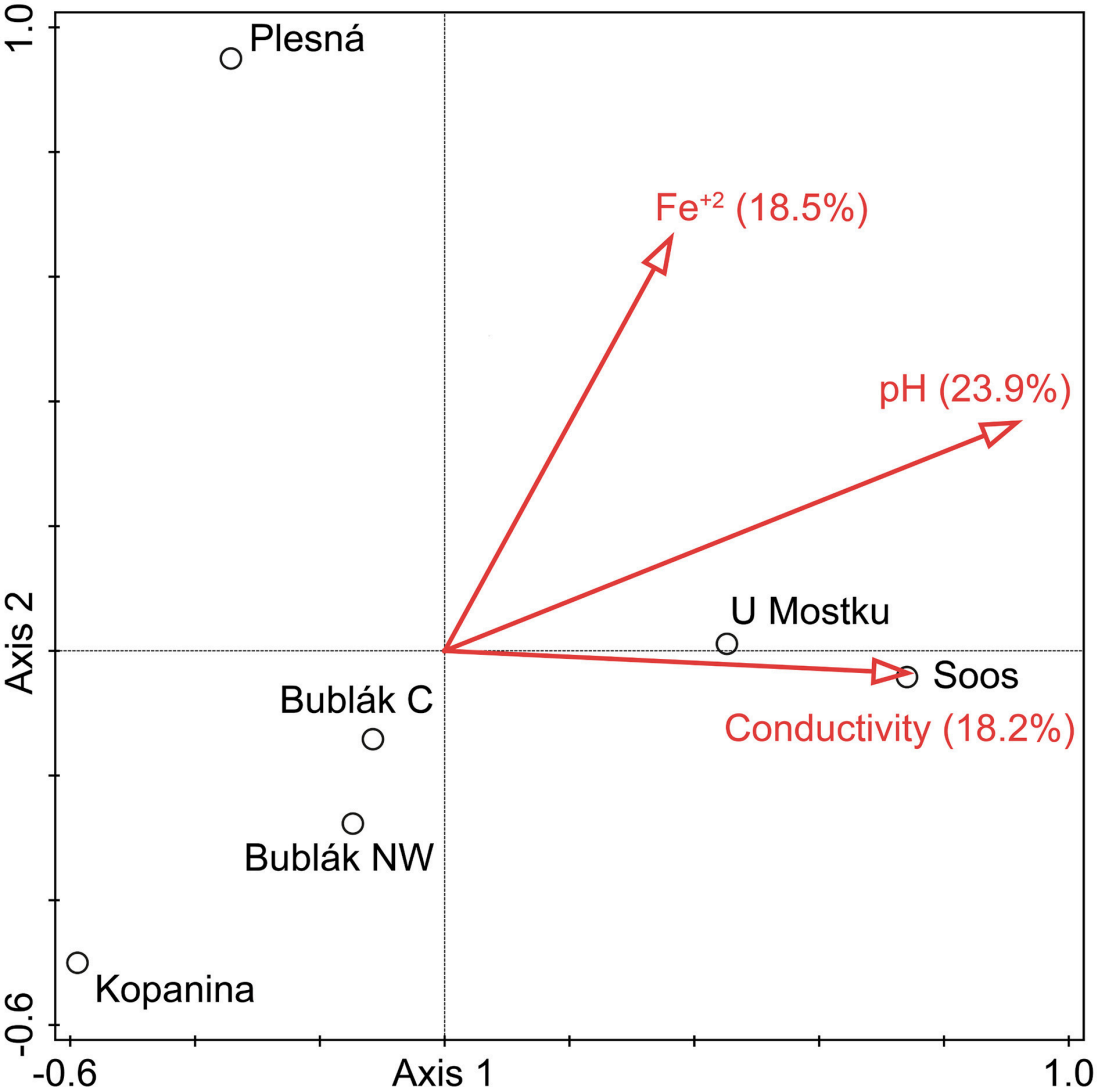
Canonical correlation analysis of the microbial composition on OTU level and environmental parameters. If the Bonferroni corrected *p*_adj_ was <0.05, a given parameter was included. Conductivity, pH and Fe^2+^ concentration explained 60.6% of the compositional variation among the investigated waters. Circles indicate sampling locations.

The relationship between microbial community structure and the environment was examined by canonical correlation analysis (Figure 6). The pH value (23.9%), Fe^2+^ concentration (18.5%), and conductivity (18.2%) were the optimal subset of environmental parameters to explain the community structure of the investigated waters (all *p*-values < 0.05). Thereby, the strength of influence of given parameters on the sites differed greatly. The community structures from both U Mostku and Soos correlate positively with pH and conductivity. The Fe^2+^ concentration showed a strong positive correlation with the community structure of the mineral spring water (Plesná). On the contrary, a moderate negative correlation with the communities from Bublák C and Bublák NW was detected. Both Fe^2+^ concentration and pH had a strong negative correlation with the microbial composition from the Kopanina water. Correlation coefficients for the most abundant taxa and all measured environmental parameters were calculated. Thereby, *Sideroxydans* showed a strongly negative (*p* = 0.03, *R*^2^ = 0.69) and *Sulfuricurvum* a strongly positive (*p* = 0.03, *R*^2^ = 0.71) correlation with the pH value.

To examine possible correlations of gene abundances to the environmental parameters, the Pearson correlation coefficient was calculated. A strong positive correlation between dissolved organic carbon and the abundance of 16S rRNA genes (*p* = <0.02; *R*^2^ = 0.79) was observed. Due to low abundances, this calculation could not be performed for *dsrB* and *mcrA* genes.

## Discussion

Natural CO_2_ conduits offer a unique opportunity to investigate adaptation of microbial communities to extremely elevated CO_2_ concentrations and its side-effects. Likewise, insight into geo-bio interactions and matter cycling in a habitat interconnected with the deep subsurface can be gained. Several studies focused on hydrochemical and geophysical features of wet mofettes (Pfanz, 2008; Kämpf et al., 2013), but biological processes were so far not considered. Most knowledge on environmental microbial communities in habitats with elevated CO_2_ concentrations were either gained from dry mofettes or soils (Krüger et al., 2009, 2011; Beulig et al., 2016), Carbon Capture and Storage (CCS) sites (Morozova et al., 2010; Wandrey et al., 2011a,b; Pellizzari et al., 2016) and deep subsurface fluids (Emerson et al., 2016; Probst et al., 2017).

The present interdisciplinary survey provides insight into the hydrochemistry and complex microbial community structure and ecology of six CO_2_ influenced mofette and mineral waters of the Cheb Basin. The microbiological survey introduces a new perspective on mofettes. Community analyses revealed that mofettes are either analogs or rather windows into the deep biosphere and furthermore enable access to deeply buried paleo-sediments.

### The core community of wet mofettes in the Cheb Basin

Hydrochemical analyses revealed that the investigated sites do not only differ in terms of physico-chemical properties (pH, conductivity, temperature, ion composition, DOC) but also with respect to their water origin (δ^2^H: δ^18^O, δ^18^O: δ^34^S) (Table 1). Waters of Plesná, U Mostku, and Bublák NW are shaped through meteoric origin whereby Soos and Bublák C waters bear higher proportions of deep ground water. Most likely the differences between both Bublák sites can be explained by the rate of degassing and admixing of shallow groundwater.

With respect to the differing origins and hydrochemistry of the investigated waters, it is remarkable that a core microbial community can be defined which made up the majority of the observed taxa. In total 50.5% of all taxa (98.9% of all reads) occurred at all sites. The microbial communities were mainly composed of putative microaerophilic, anaerobic lithoautotrophic organisms, capable to fix the provided magmatogene CO_2_ and gain energy by oxidation or reduction of inorganic substrates such as iron or sulfur. The simultaneous occurrence of organotrophic species can be linked to the admixture from surrounding soil. Potential phototrophic organisms (Table S4) played only a role at the ponds from unprotected springs (Bublák and Plesná). DOC data from the ponds in Bublák and Plesná, where foliage builds large benthic and littoral layers, led to the assumption that microbial degradation of complex organic carbon is restricted under anaerobic or microaerophilic conditions. This degradation inhibiting effect of CO_2_ coupled to anoxia and low pH was also reflected in the low amounts of organic acids. Due to these conditions, the up streaming CO_2_ was already considered as primary carbon source for microbial metabolism at mofette soils (Beulig et al., 2016).

The genera *Gallionella* (13.71%), another *Gallionellaceae*-related taxon (3.06%), *Sideroxydans* (2.59%), the sulfur oxidizers *Sulfuricurvum* (9.46%), and *Sulfurimonas* (3.43%) were identified as generalists occurring at all sites in high relative abundances. At the same time, the SIMPER analysis revealed that the abundances of these taxa determine the differences between the microbial communities of the surface and subsurface waters (Table S3). Similar taxonomic and physiological groups (e.g., *Gallionellales*, sulfur oxidizers) were dominant in deep, CO_2_ affected subsurface fluids (Emerson et al., 2016). Therefore, microbial iron and sulfur oxidation might play an important role in CO_2_ influenced aquatic subsurface systems. In contrast, only a few specialists such as *Hadesarchaea* were site-specific. The identification of such a copious core community (98.3–99.62% of total counts at a given site) could indicate connectivity through a deep aquifer between the investigated sites, despite the observed different geochemical properties of the waters and the distances of more than 15 km. Accordingly, multivariate statistics showed that the differing, site-specific environmental parameters (e.g., Fe^+2^ concentration, conductivity) did not determine the occurrence of most taxa, but rather shifted the community composition and relative abundances of certain organisms (Figure 6).

### Quantity and diversity of microorganisms with respect to environmental parameters

Our results indicate that a high CO_2_ partial pressure in aquatic habitats does not necessarily cause low microbial abundances, since the autochthonous microorganisms are well adapted to these conditions. The observed abundances for 16S rRNA genes (qPCR, Table 2) in the investigated waters are in compliance with studies in similar, but not CO_2_ affected habitats, for instance ground water or water from granite systems (Griebler and Lueders, 2009 and references therein). The differences in microbial abundances between the investigated waters could be explained by the varying amount of allochthonous influx of substrates and organisms. Comparative analyses of the water phases and the surrounding sediments indicated a rather different microbial community (Figure 3). The same argument explains the higher microbial diversity in surface waters compared to the subsurface originated waters, namely U Mostku and Soos.

The influence of elevated CO_2_ concentrations coupled with changes of pH as well as high concentrations of iron mainly shaped the community structure and abundances of specific taxa (Figures 1, 4). In addition, the Fe^2+^ concentration showed a strong negative correlation with the diversity. In the case of the investigated waters, higher Fe^2+^ concentrations led to a decreased diversity and an increased abundance of iron oxidizing bacteria, especially of *Gallionella*-related organisms. The only exception to this observation was the water from Soos. Even though the Fe^2+^ concentration was on par with the one measured in the water from Plesná, the very low amount of oxygen and high salinity inhibits the growth of *Gallionella-*related organisms (McBeth et al., 2013). Generally, decreased bacterial community richness, evenness and diversity have been reported for dry mofette sites (Sáenz de Miera et al., 2014). Our results indicate that an active fluid flow, driven by the uprising CO_2_, provides better substrate supply and enables a higher microbial diversity in comparison to dry mofette sites.

In order to interpret the ecological function of the detected microbial communities, we assigned the functional potential of single OTUs based on a literature review. Despite the fact that this approach cannot guarantee completeness and accuracy, we can observe interesting patterns in respect to the major matter cycles. The occurrence of iron-oxidizing bacteria was a common feature across all investigated waters. They were especially abundant in surface waters (Plesná, Bublák Central, Bublák NW, Kopanina). Besides smaller quantities of *Sideroxydans*- and *Ferritrophicum*-related organisms, *Gallionella* was the most abundant genus related to iron cycling. Organisms affiliated to the genus *Gallionella* are chemolithoautotrophic, microaerophilic iron-oxidizers, which increase their biomass production with increased carbon dioxide concentrations (Hallbeck et al., 1993). *Gallionella*-related organisms are common in various freshwater habitats where ferruginous water comes in contact with oxygen, for instance spring water or groundwater. Their habitat needs a low redox potential, a pH 6–7.6, low oxygen concentration (0.1–1 mg L^−1^), CO_2_ > 20 mg L^−1^ and high amounts of Fe^+2^ (5–25 mg L^−1^) (Hanert, 1975). Further, it is known that *Gallionella* stimulates the formation of iron oxides at the early stage of clogging (Wang et al., 2014), thereby affecting injection or re-injection rates of wells. Interestingly, the most acidic surface water (Kopanina) showed no predominance of *Gallionella*, but a shift to *Sideroxydans*- and especially acidophilic *Ferritrophicum*-related organisms. Metagenomic studies on deeply sourced CO_2_-enriched fluids showed that the metabolic capability of nitrate/nitrite reduction could be present in *Gallionellales*-related organisms (Emerson et al., 2016), increasing the fitness in a microaerophilic/anaerobic environment. CO_2_ affected surface waters could represent oxic-anoxic transition zones, which provide beneficial conditions for the growth of iron oxidizing bacteria. Both the acidification of the respective water (pH 4.2–6), and the low oxygen concentrations, due to a high CO_2_ partial pressure, promote microbial iron oxidation in such a habitat. This is reflected in the high abundances of iron oxidation related genera in the studied surface waters, which made up to approximately 20% of the microbial communities.

Another common feature of the investigated waters is the occurrence of sulfur cycle related organisms. Especially the waters from U Mostku and Soos showed high abundances of microorganisms involved in sulfur cycling. These results coincide with the isotopic ratio of the dissolved sulfate of the waters, which indicate bacterial oxidation of sulfur and reduced sulfur compounds to sulfate. The largest fraction of sulfur cycling related taxa is associated with sulfur oxidation. The genus *Sulfuricurvum* was highly abundant, making up approximately 20% of the microbial communities from the subsurface waters (U Mostku, Soos). *Sulfuricurvum*-related organisms are described as anaerobic/microaerophilic and sulfur-oxidizing chemolithoautotrophs, which can use a variety of electron acceptors (e.g., oxygen, nitrate) and electron donors (e.g., elemental sulfur, sulfide, thiosulfate) (Kodama and Watanabe, 2004) and are widely distributed in subsurface habitats (e.g., Engel et al., 2003). Besides sulfur oxidation related organisms, sulfate reducing bacteria (SRB) were present in CO_2_ influenced mofette and mineral waters, which made up to 0.5–3.8% of the total microbial community. Sulfate-reducing bacteria obtain energy by coupling the oxidation of organic compounds (e.g., alcohols, organic acids) or H_2_ to the reduction of sulfate, generating hydrogen sulfide. The largest fraction of SRB was observed in the water containing the highest concentrations of acetic acid (U Mostku). It is assumable, that the limited availability of low molecular organic acids (e.g., acetic acid) due to a restricted degradation of organic material under anoxic conditions could inhibit the growth of SRB. At least 24 different taxa related to sulfate reduction were found. The most abundant genus *Desulfosporosinus* is, according to several studies (e.g., Senko et al., 2009; Sánchez-Andrea et al., 2015), associated with acidic environments. Strictly anaerobic sulfate reduction was also observed at dry mofettes, which most probably was enabled by the absence of oxygen and the presence of organic carbon and geogenic carbon dioxide and hydrogen (Beaubien et al., 2008; Frerichs et al., 2013). We conclude that both, wet and dry mofettes represent, depending on the availability of low molecular weight organic acids, suitable habitats for sulfate reducing bacteria.

Regarding the nitrogen cycle, several pathways were indicated by the taxonomic analyses. In total, 17 bacterial and archaeal taxa (2% of all reads) implicated in nitrification were found. Among the ammonia-oxidizing taxa, *Nitrosomonadaceae* and interestingly the archaeon “*Candidatus* Nitrosotalea” were most abundant. Recently, Lehtovirta-Morley et al. (2016) could show that the obligate acidophilic ammonia oxidizer “*Candidatus* Nitrosotalea devanaterra” contains genes encoding both a predicted high-affinity substrate acquisition system and potential pH homeostasis mechanisms absent in neutrophilic species. Therefore, previously proposed mechanisms used by ammonia-oxidizing bacteria for growth at low pH are not essential for archaeal ammonia oxidation in acidic environments. Low abundances of taxa involved in the anaerobic oxidation of ammonia (ANAMMOX) were also found. Besides “*Candidatus* Anammoximicrobium,” also “*Candidatus* Brocadiaceae” was detected in the Kopanina and U Mostku waters. Whereby ubiquitous distributed nitrite-oxidizing bacterial (NOB) genera, such as *Nitrospira* and *Nitrobacter*, were found in low relative abundances, “*Candidatus* Nitrotoga”-like bacteria (Alawi et al., 2007) were dominating the NOB community. Again, most likely the pH is the crucial environmental parameter controlling the distribution pattern of the microbial community. Community analyses of nitrifying biofilms revealed a coexistence of *Nitrospira* and “*Candidatus* Nitrotoga,” and it is hypothesized that a slightly acidic pH in combination with lower temperatures favors the growth of the latter (Alawi et al., 2009; Hüpeden et al., 2016). We conclude that “*Candidatus* Nitrotoga” is not only from high importance in permafrost regions, rivers or wastewater plants, but in general in microaerophilic habitats facing mean temperatures below 18°C.

In addition to iron, sulfur and nitrogen cycling microorganisms, archaea related to methane cycling were found. These methanogenic archaea showed, in relation to the whole community, low abundances. Only the shallow subsurface water (U Mostku) had major portions in methanogenic archaea (*Methanoregula*) in relation to the total community. *Methanoregula* is an acidophilic methanogen, which utilizes hydrogen and CO_2_ but no organic compound for methanogenesis (Brauer et al., 2011), and is widely distributed in different environments across the globe (Wen et al., 2017; Yang et al., 2017). Furthermore, acidophilic *Methanoregulaceae*-related microorganisms have been observed before at dry mofette sites (Beulig et al., 2015). *Methanoregula* depends on acetate for growth, which was mainly present in the shallow subsurface water U Mostku (1.6 mg L^−1^; Table 1). Therefore, we hypothesize that *Methanoregulaceae* play an important role in carbon cycling in the anoxic environment of wet and dry mofettes. In contrast, Beaubien et al. (2008) observed an absence of hydrogen-dependent methanogenesis at mofette sites and suggested this pathway is inhibited in high CO_2_ environments due to a lack of hydrogen. The abundance of this genus militates against a putative inhibition of hydrogen dependent methanogenesis in such a habitat. Despite the abundance of methanogenic archaea, observed methane fluxes were very low and the isotopic data indicated a thermogenic origin of the methane. It should be considered that a mixture of methane formed biogenically near the surface (δ^13^C ≈ −80‰) and highly ^13^C-enriched methane originating from the upper mantle (^13^C ≈ −15‰; e.g., Etiope and Sherwood Lollar, 2013) could result in the measured δ^13^C-methane values (Bräuer et al., 2005). Therefore, microbial methane production cannot be completely ruled out, especially because methanogens were detected in each of the investigated mofette waters.

### Interconnection of mofette waters with paleo-sediment and the deep biosphere

Our results, based on hydrochemical and isotopic analyses as well as Illumina 16S rRNA gene sequencing, not only provide first evidence that the surface waters are interconnected via the up streaming fluids with deep subsurface paleo-sediment but in addition they are linked to the deep biosphere.

*Hadesarchaea* (formerly South-African Gold Mine Miscellaneous Euryarchaeal Group, SAGMEG) were solely found in the Soos mineral water (0.4%). This group is described as metabolically versatile and shares several physiological mechanisms with strict anaerobic *Euryarchaeota* (Takai et al., 2001; Parkes et al., 2005; Biddle et al., 2006). They are prominent members of the deep subsurface biosphere and occur both in terrestrial and marine environments, including hot springs. Based on genomic reconstructions, it is assumed that *Hadesarchaea* are mediating key geochemical processes which allow them to successfully inhabit the deep subsurface (Baker et al., 2016). They show metabolic characteristics, such as CO and H_2_ oxidation (or H_2_ production), with potential coupling to nitrite reduction to ammonia (DNRA). *Hadesarchaea* possess central carbon metabolic (C1 pathway) genes, which may be used for carbon fixation (Baker et al., 2016) and thereby fit to the conditions in the mineral water of Soos.

Besides of *Euryarchaeota* involved in methane cycling, we identified *Bathyarchaeota* (formerly Miscellaneous Crenarchaeota Group 1, 6, 7/17 and 15). These organisms have been found in marine sediments and deep aquifer waters and seem to be capable of acetogenesis and methane metabolism (Kubo et al., 2012; Evans et al., 2015; He et al., 2016). Sequences of this group not only have been found in the mineral fluid with a deep origin (Soos, 0.82%), but also in smaller quantities in the CO_2_ affected surface waters. In the extreme environment of active CO_2_ conduits, these organisms could represent another player involved in anaerobic carbon cycling. Furthermore, this finding is, in addition to the occurrence of *Hadesarchaea*, another indicator for the connection between surface and deep subsurface habitats—or reflects deep biosphere conditions in a surface habitat.

The carbon/hydrogen isotope ratios of methane of the investigated sites indicated a thermal gas, similar to the methane of the high saline brine analyzed in the German Continental Deep Drilling Project (KTB) pilot borehole, which is located approximately 50–80 km away from the investigated mofette region (Faber, 1995; Möller et al., 1997; Lippmann et al., 2005). Both, the methane of the KTB borehole and the methane of the free gases from mofettes of the Cheb Basin could be linked to the same or similar marine-influenced Permo-Carboniferous sedimentary basin.

The finding, that the mofette waters are potentially interconnected to deeply buried marine paleo-sediment, is supported by the observation of more than 50 taxa known to be obligate “marine” organisms, which were to our best knowledge, so far not observed in shallow terrestrial Central European ecosystems. The highly saline deep waters might serve sufficient conditions for the microorganisms to survive and proliferate; however, this explanation does not provide an adequate answer of their origin. Combining the results of the microbiological and isotopic water/gas analyses, the saline fluids are most likely allochthonous and migrate from the sediments of marine origin of one of the Permo-Carboniferous basins into the vicinity, for instance the Weiden Basin, located approximately 50–80 km SW of the Cheb Basin. Originally, this hypothesis was formulated for the highly saline basement brines of the KTB-pilot hole (Faber, 1995; Möller et al., 1997, 2005; Lippmann et al., 2005). Möller et al. (2005) assumed that during the Upper Cretaceous uplift of the adjacent basement (>2,000 m according to Wagner et al., 1997) the formation waters from Triassic to Carboniferous strata penetrated along the Franconian lineament NE- wards into metamorphic sequences of the crystalline basement of the Bohemian Massif. Infiltration of the brine might have occurred about 70 Ma concurrent with the uplift of the Bohemian Massif.

The microbial community of the investigated waters consists of a mixture of species which originate from the deep subsurface (saline thermal waters and paleo-sediments) and the surface (shallow groundwater aquifers filled with meteoric water and surrounding sediments). The emanating CO_2_-rich fluids cross and interconnect these habitats. Our analysis of the carbon/hydrogen isotope ratios of methane linked the origin of the fluids to a marine-influenced sedimentary basin. Therefore, marine species might originate from deep saline aquifers or marine sediments from the Weiden Basin. These marine species possibly persisted over millions of years in great depth and are transported to the surface via the emanating waters. These observations strengthen our assumption that wet mofettes are not only partly analogs but moreover windows to the deep biosphere and paleo-sediment.

However, it cannot be ruled out that the particular geochemical setting of the fluids enable marine species, introduced through meteoric waters, to proliferate. In 2016, a drilling campaign was conducted at one of the mofette fields in the Cheb Basin (Bussert et al., 2017). The 108.5 m deep drilling into a CO_2_ conduit will provide further insights into microbial processes and the origin of these microorganisms.

## Conclusion

The present interdisciplinary survey on wet mofettes improves our understanding of microbial life under strongly elevated CO_2_ concentrations. The results from hydrochemical analyses, from isotopic signatures of key elements and from the 16S rRNA gene profiling unveil a new perspective on mofette research. The results indicate that mofette waters in the Cheb Basin are connected with the deep subsurface, specifically paleo-sediments and the deep biosphere. Therefore, mofettes provide access to deeply buried geo-bio-archives. Further on, it has to be considered that the mofette waters are at least in one direction interconnected. This argument is strengthened by the observation that among all investigated sites a large fraction of generalists and only a very small fraction of site specific specialists was found. Moreover, the described community in surface waters from mofettes of central Europe is in large proportions similar to the deep biosphere of geysers and marine thermal vents, such as black smokers. Mainly the influence of elevated CO_2_ concentrations coupled with changes of pH as well as varying concentrations of iron shaped the community structures and abundances of specific taxa. The gained knowledge and especially the community shift to taxa well adapted to low pH might be useful regarding geo-engineered systems (e.g., geothermal energy, wastewater treatment, drinking water reservoirs, regional spas and carbon or hydrogen subsurface storage facilities).

## Author contributions

PK performed DNA extractions and prepared genetic analyses and gene quantifications, and contributed writing the present manuscript. HK sampled during the field campaign, performed geochemical analyses and contributed writing the manuscript. FH performed bioinformatic analyses of the sequence data set and contributed writing the manuscript. QL was involved interpreting the results of the community study and designed figures. AV performed isotopic analyses and interpreted the results. DW provided important financial and technical support and supported the writing of the manuscript. MA designed the study and supervised DNA extractions and genetic analyses and led the writing of the present manuscript. All authors have taken part in the manuscript revisions and agreed with its scientific content.

### Conflict of interest statement

The authors declare that the research was conducted in the absence of any commercial or financial relationships that could be construed as a potential conflict of interest.
