# Supplementary material for: Microbiological and Geochemical Survey of CO2-Dominated Mofette and Mineral Waters of the Cheb Basin, Czech Republic
Source: Front Microbiol. 2017 Dec 11;8:2446. doi: 10.3389/fmicb.2017.02446 (PMC5732176; doi:10.3389/fmicb.2017.02446)
Supplement: Supplementary file 5 [file Image1.PDF]

SUPPLEMENTARY MATERIAL

Microbiological and geochemical survey of CO_2-dominated mofette and mineral waters of the Cheb Basin, Czech Republic

Patryk Krauze[1], Horst Kämpf[2], Fabian Horn[1], Qi Liu[1], Andrej Voropaev[3], Dirk Wagner[1] and Mashal Alawi[1*]

[1]GFZ German Research Center for Geosciences, Section Geomicrobiology, Telegrafenberg, Potsdam, Germany

[2]GFZ German Research Center for Geosciences, Section Organic Geochemistry, Telegrafenberg, Potsdam, Germany

[3]Hydroisotop GmbH, Woelkestraße. 9, D-85301 Schweitenkirchen, Germany

Figures

Fig. S1: Rarefaction analysis of investigated mofette waters and related sediments, and mineral waters using the Shannon's H index. BNI = Bublák NW, BESI = Bublák Sediment, BZI = Bublák C, KI = Kopanina, PI = Plesná, PSEI = Plesná Sediment, SI = Soos, SSEI = Soos Sediment, UMI = U Mostku

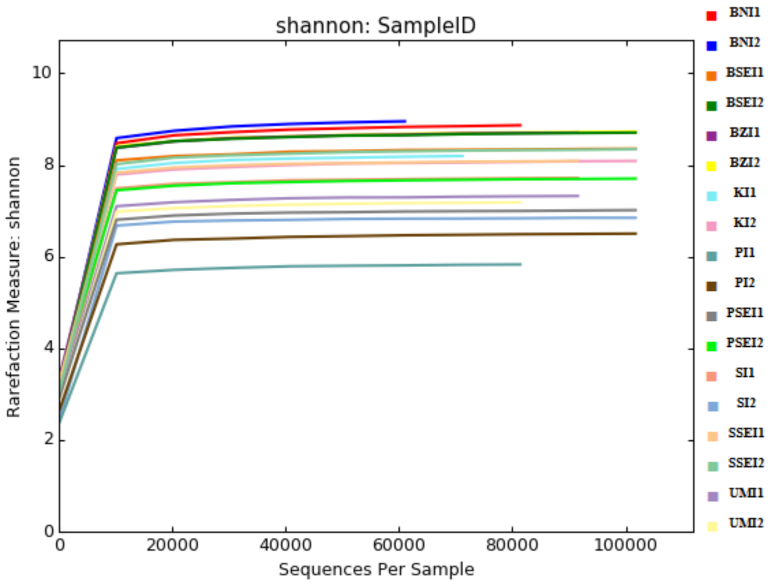

Fig. S2: Box plot analyses of the sequence data set. The distance (in terms of unshared OTUs) within replicates is significantly lower than the distance between the sites.

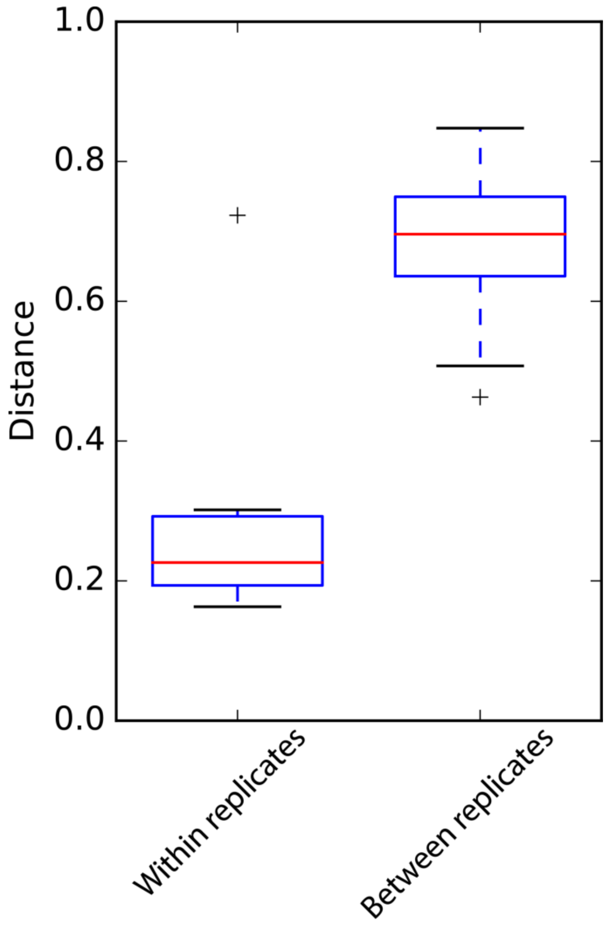

Tables S1-S4 (separate CSV files)

Tab. S1 Number of reads after quality filtering and deletion of chimeric sequences for each sequenced sample.

Tab. S2 Observed species and Shannon's H indices of each site with different rarefaction thresholds.

Tab. S3 SIMPER analyses comparing subsurface and surface waters

Tab. S4 OTU distribution of investigated mofette waters and related sediments, and mineral waters.
